# Intubation difficulty scale contributors and time delay in clinical practice

**DOI:** 10.1097/MD.0000000000028724

**Published:** 2022-01-28

**Authors:** Ting-Wei Kang, Jung-Der Wang, Yi-Seng Tsai, Chung-Ren Lin, Chia-Chih Alex Tseng

**Affiliations:** a Department of Anaesthesiology, National Cheng Kung University Hospital, College of Medicine, National Cheng Kung University, Tainan, Taiwan; b Department of Public Health, National Cheng Kung University Hospital, College of Medicine, National Cheng Kung University, Tainan, Taiwan; c Department of Anaesthesiology, Ditmanson Medical Foundation Chia-Yi Christian Hospital, Chia-Yi, Taiwan.

**Keywords:** airway management, difficult intubation, duration of successful intubation, IDS, intubation time, perceived difficulty of intubation

## Abstract

The intubation difficulty scale (IDS) includes 7 contributors that provide a comprehensive assessment of difficult intubation. However, the effect of each contributor is unclear, and the scale has not been revalidated recently and has not been validated in orient. This study determined the duration of successful intubation (DSI) for each of these 7 contributors.

The patients were intubated by attending anesthesiologists. The duration and other data were recorded by 2 research assistants. Anaesthesiologists reported the IDS and their perceptions. A linear mixed-effects model with a DSI was constructed using IDS factors.

In total, 1095 patients were enrolled. The average DSI was 23.9 ± 21.8 seconds (37.1% IDS = 0). All 7 factors were independently associated with duration, with the exception of vocal cord adduction. The best model was as follows: DSI (in seconds) = 15.2 + 31.1 (number of additional attempts) + 26.2 (number of additional operators) + 11.4 (number of alternative techniques) + 7.9 (increased lift force) + 4.9 (external laryngeal pressure) + 3.5 (Cormack grade 1). The mixed models were similar except for the regression coefficient for the number of alternative techniques that decreased from 11.4 to 6.9 seconds.

We confirmed that each IDS contributor affects the DSI and validated a prediction model with 6 IDS contributors. This prediction model may facilitate the development of strategic plans for critical airway management. Furthermore, it could improve simulations and monitor learning progress and help provide valuable feedback.

## 1 Introduction

Tracheal intubation, first introduced in 1880, has saved many lives. Intubation techniques have improved and instances of difficult intubation (DI) have decreased dramatically; however, unanticipated DI crises still occur.^[1–3]^ To improve the quality of airway management, the human factor was emphasised, and crisis management simulations were suggested.^[4]^ However, the definition of DI varies, with multiple attempts,^[5]^ more than 3 attempts,^[6]^ or impossible intubation by experienced anaesthesiologists^[5,6]^ being some of the criteria. These do not consider the entire range of difficulty from easy to impossible intubation or rare incidents (1/1000–0.1/1000). Learners have simply failed or succeeded when practicing intubation, with no common language employed for the progress of the skill. Therefore, monitoring the educational progress of learners^[7]^ and providing feedback on airway management is difficult.

The intubation difficulty scale (IDS)^[8]^ centres on the experience and perception of difficulty by the operator, and the actual physical obstacles and continual data are useful to present the complexity of tracheal intubation.^[8,9]^ Moreover, the IDS included 7 factors related to intubation difficulty. The scale was initially validated with a small number of cases (315)^[8]^ and by doctors with more than 2 years of experience in terms of intubation time (correlation *τ* = 0.48; *P* < .0001) and visual analog scale (VAS) of difficulty (correlation *τ* = 0.63; *P* < .0001), and the researchers investigated the correlation between the IDS score and VAS or duration. Moreover, they set every factor to have an equal impact. In clinical settings, vocal cord adduction or laryngeal pressure may not be sufficient to motivate a change in the tool or doctor. Revalidation data, 4 years after the initial validation, also did not report intubation time.^[10]^ This limited IDS data has been used to improve practice in clinical and crisis simulation education. Finally, no detailed analysis has been conducted in an Asian population. Improving the usage of the IDS in clinical education, improving intubation practice in general, and defining simulation settings to establish a comprehensive evaluation scale for each clinical step are imperative. An IDS is considered a comprehensive score for tracheal intubation. However, revalidating the IDS and considering the effect of the 7 contributing factors on DI may help to evaluate the learning progress in crisis management settings.

The duration of successful intubation (DSI) is a concrete objective outcome for tracheal intubation, which is commonly used for clinical comparison and training.^[7,11–14]^ Clinically, a novice requires 60 seconds to complete intubation after practice, and 30 seconds is necessary for easy cases. Additionally, prolonged DSI is associated with major adverse outcomes or death.^[15]^ However, an independent person must record DSI. This is not always feasible in daily practice, nor is it convenient for clinical care monitoring and evaluation of clinical progress. Identifying the role of each contributor on DSI may provide a formalised algorithm outlining the order of useful steps for evaluating learning progress and facilitating the development of crisis strategies and simulations.

A comprehensive analysis of the IDS in terms of time and perceived difficulty using real-world conditions and highly experienced anesthesiologists performing tracheal intubation may improve the usefulness of the IDS when evaluating novice doctors and in simulations.

Herein, we introduce a comprehensive validation analysis to validate the IDS with DSI and the perceived difficult intubation (PDI) for highly experienced anaesthesiologists (with more than 6 years of full-time practice and 1000 intubations per year). We analysed the effects of varying degrees of IDS and the effect of each contributing factor on DSI using a multiple regression model. A mixed-effect model was applied to clarify the personal effects of 12 highly experienced doctors.

## 2 Materials and methods

After approval was granted by the Ethics Committee of the Ditmanson Medical Foundation, Chia-Yi Christian Hospital (IRB No. 097028), we obtained written informed consent from each patient. The ethics committee also required oral consent from the surgeons responsible for the operations. Consecutive adult patients (≥20 years old) from the surgery, urology, gynaecology, and orthopaedic departments who were scheduled to undergo general anaesthesia were invited to participate in the study and were interviewed by a research assistant. Patients who had received tracheal intubation under direct laryngoscopy with a #3 Macintosh blade were selected. Patients scheduled for general anaesthesia with double-lumen bronchial intubation, those with upper airway pathology (eg, maxillofacial fractures or head or neck deformities) or cervical spine fractures, or those who were initially intubated using other equipment were excluded from the study. To ensure measurement quality, 4 patients were enrolled each day during regular working days from November 2011 to December 2014. Thus, data from 81% of all eligible patients during the study period were collected. They were enrolled in a prospective observational study of anaesthesia-related outcomes in a tertiary hospital located in southern Taiwan.

One day before the operation, trained research assistants abstracted demographic and physical data, including sex, age, body mass index, and the American Society of Anesthesiologists Classification, from the participants’ medical records and performed a routine airway physical examination. The airway examination provided discrete data for cervical spine mobility (yes/no), dentition (yes/no), neck radiation or mass (yes/no), thyromental distance (in centimetres), jaw protrusion (in millimetres), and the Mallampati oropharyngeal classification (as modified by Samsoon and Young).^[16,17]^

After induction, the attending anesthesiologists reported the direct-view Cormack grade,^[18]^ adduction of the vocal cord, and whether increased lifting strength was required for intubation. The assistants recorded and measured other variables, including the number of additional intubation attempts required, number of additional operators required, and number of alternative intubation techniques used. The glottic view was defined using the Cormack grade (grade 1 = 0; grade 2 = 1; grade 3 = 2; grade 4 = 3),^[18]^ lifting force applied during laryngoscopy (1, if increased force was applied; 0, if no increased force was applied), whether external laryngeal pressure was required to improve the glottic view (yes/no), and the position of the vocal cords during intubation (1 if adducted; 0, if abducted or not visible) was also recorded. The sum of the scores for the 7 aforementioned variables represents the total IDS score. An IDS grade was assigned for that score,^[5]^ where 0, 1 to 5, >5, and ∞ indicated easy, slight difficulty, moderate to major difficulty, and impossible intubation or failure of tracheal intubation, respectively.^[8]^ In addition, the subjective PDI was reported on a 4 point Likert scale: grade 1 = “easy,” grade 2 = “not easy,” grade 3 = “difficult but possible,” grade 4 = “extremely difficult, without confidence of successful intubation.” Moreover, a research assistant recorded the duration of each intubation attempt, which was then summed to obtain the DSI. The duration of each tracheal intubation attempt was defined as the time taken from insertion of the blade between the teeth to the time the tracheal tube cuff was inflated or the blade was removed from the mouth. If more than 1 attempt was required, the patient received bag-and-mask oxygenation between attempts. During surgery, each patient hemodynamic parameters and the medications they received were recorded.

### 2.1 Statistical analysis

The sample size was calculated as the required sample size for an incidence rate of 6.3% for IDS > 5 based on the original report^[8]^ with a marginal error not exceeding 2% with a 95% confidence level. The calculated sample size was 567 patients. Continuous variables are presented as mean ± standard deviation, and categorical variables are presented as count and percentage. Multiple linear regression modelling was used to estimate the average delay caused by individual IDS factors. Regression modelling was also performed with DSI by subtracting the mean of with an IDS score of 0 (easy intubation) to test the robustness of the original model. A mixed-effects model, with the same intubation performer assumed as the random effect, was also employed to control for the potential effect of autocorrelation of the 12 anaesthesiologists. A similar model was applied to the subjective outcome of the PDI of practice doctors. The 12 DI prediction factors were entered into the model to test the individual and combined effects of IDS factors and common DI prediction risk factors on DSI. All the statistical analyses were 2-sided and evaluated at a significance level of 0.05. All analyses were performed using SPSS (version 17.0, SPSS Inc., Chicago, IL). The generalised linear mixed model was calculated using SAS (SAS 9.4; SAS Institute Inc., Cary, NC).

## 3 Results

The demographic and preoperative DI assessment data of 1095 patients are summarised in Table 1. All of them completed complete data collection. The majority of patients (715% or 65.3%) were female, and 1045 patients (95.4%) were intubated after only 1 attempt. In total, 406 (37.1%) and 19 (1.8%) patients had IDS scores of 0 (easy) and >5 (moderate to major difficulty), respectively. None of the patients had an IDS score of ∞ (impossible intubation; Fig. 2).

**Table 1:** Demographic and clinical characteristics of the participants before surgery.

	N = 1095
Age (yr)	54.3 ± 15.0
Body mass index (kg/m^2^)	26.7 ± 6.6
Female (%)	715 (65.3)
American Society of Anesthesiologists (ASA)	
I	197 (18.0)
II	845 (77.2)
III	53 (4.8)
Surgery	
Thyroid	175 (16.0)
Breast	160 (14.6)
Cardiovascular	9 (0.8)
Abdominal	
Upper	123 (11.2)
Lower	97 (8.9)
Bariatric	133 (12.1)
Laparoscope	170 (15.5)
Oesophageal	3 (0.3)
Orthopaedic	121 (11.1)
Spine	101 (9.2)
Superficial	3 (0.3)
Airway assessment tests	
Neck circumference (cm)	36.9 ± 4.0
Thyromental distance (cm)	7.6 ± 1.8
Mallampati score	
1–2	549 (50.2)
3–4	545 (49.8)
Cervical spine mobility (limited)	429 (39.2)
Dentition (Yes)	909 (83.0)
Neck radiation or mass (Yes)	82 (7.5)
Jaw protrusion limited (Yes)	460 (47.8)
History of snoring (Yes)	614 (56.1)
Presence of facial hair (Yes)	2 (0.2)

Mean ± SD or number (%).

**Figure 1:**
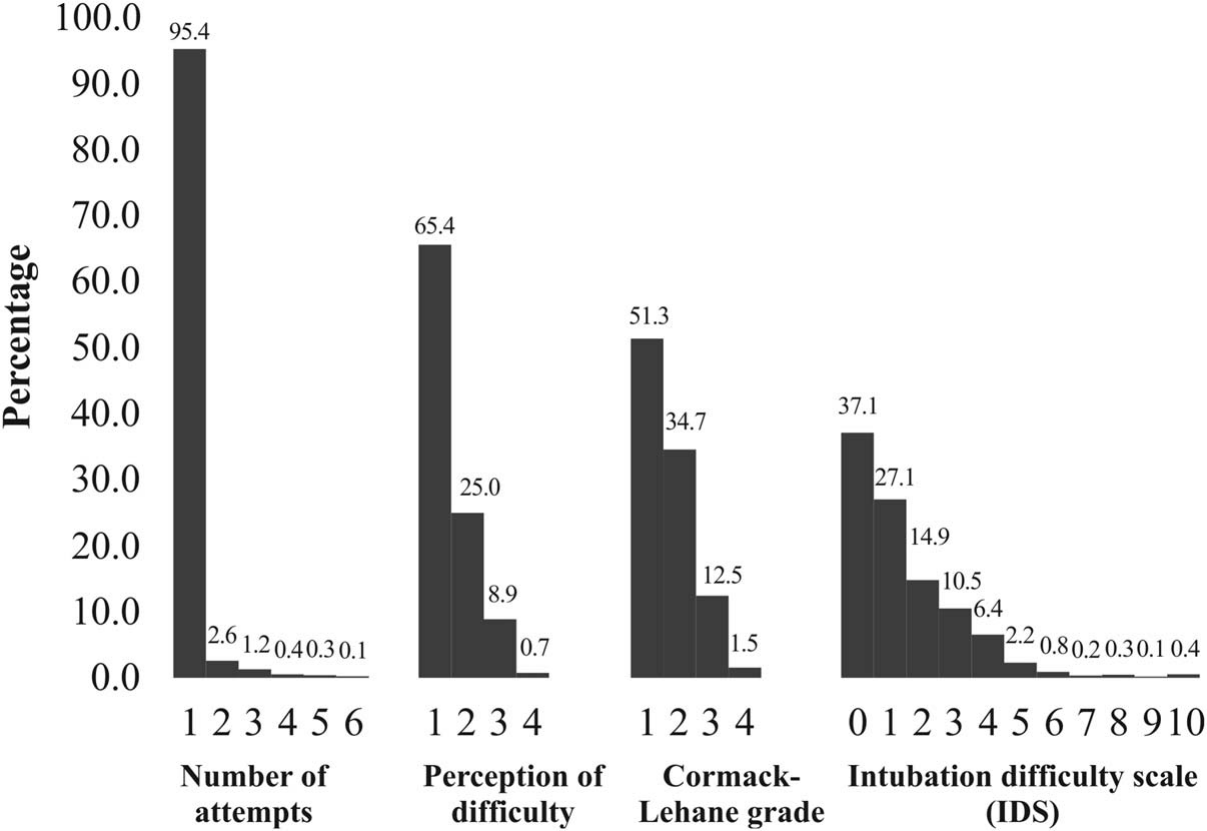
Frequency distributions of common factors used for predicting tracheal intubation outcomes for 1095 participants.

Nine patients (0.8%) required more than 3 attempts (Fig. 1), and 58% (7/12) of these attempts involved a video apparatus (optical stylet [4] and video blade [3]). The classical Macintosh blade was selected after the first attempt in 91.8% (45/49) of patients and 73.7% (14/19) after the second failure. A total of 238 (21.8%) patients required increased lifting force, and 154 (13.9%) patients had Cormack grade ≥III (Fig. 1). In total, 445 (40.6%) patients required external pressure to the larynx, and 19 (1.7%) patients were intubated with vocal cord adduction. Less than 1% of patients required an additional doctor or alternative technique for successful intubation, and 105 patients had a PDI with a Likert scale score of ≥3 (9.6%; Fig. 1).

**Figure 2:**
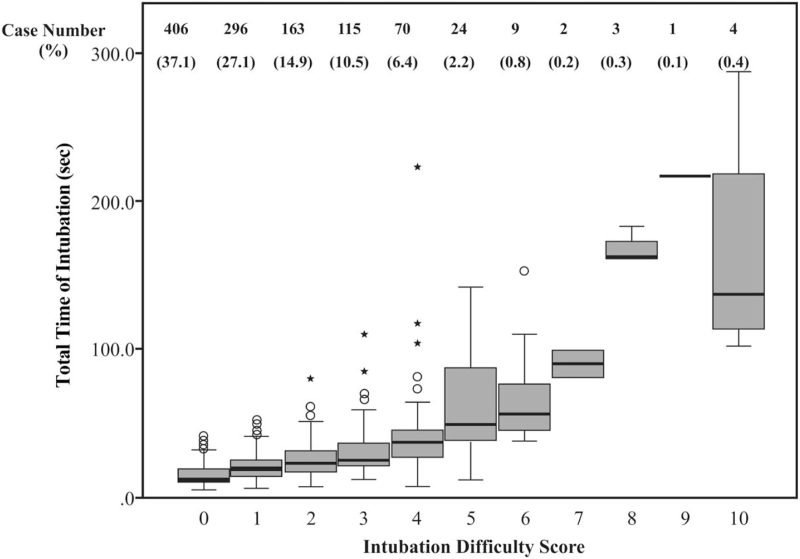
Correlation between total time of intubation and intubation difficulty scale. The bold-typed line in each box represents the duration of successful intubation for patients under a specific IDS score, with the median in the centre and the upper and lower bounds (75% upper quartile, 25% lower quartile). The numbers and percentage on the top represent those of patients under each IDS score. IDS = intubation difficulty scale.

The mean duration of first intubation attempt and DSI were 21.4 ± 12.1 and 23.9 ± 21.8 seconds, respectively. IDS scores ranged from 0 to 10 (Fig. 2). The mean DSI in “easy” patients (IDS = 0) was 15.0 ± 7.0 seconds. A total of 704 (64.6%) patients had an IDS of 0 or 1, with a DSI of <60 seconds (5–51 seconds).

All patients with Cormack grade 1 or 2 (955) were intubated successfully in fewer than 3 attempts and with an IDS score of ≤5 (easy or slight difficulty). In total, 6/137 (4.3%) patients with Cormack grade 3 and 2/17 (11.8%) patients with Cormack grade 4 required more than 3 attempts; 13/137 (9.5%) and 4/17 (23.5%) patients with Cormack grades 3 and 4, respectively, had an IDS score >5. Moreover, intubation was successful in 77.3% (115/154) of patients with Cormack grade ≥3, intubation was successful. In 51 patients, more than 1 attempt was required, with the Macintosh blade being used in 46 of them. In the second attempt, 28/46 (64%) intubations were successful, and 9/12 (75%) were successful during the third attempt with the same blade.

The mean DSI increased along with the IDS score, from 15.0 (IDS = 0) to 166.0 seconds (IDS = 10; Fig. 2). A significant correlation was observed between DSI and IDS scores and between DSI and Cormack grade, with correlation coefficient of 0.67 (*P* < .001) and 0.43 (*P* < .001), respectively. The multiple linear regression models constructed for DSI with IDS factors as independent variables revealed that 6 of the 7 factors were included in the final model as follows: DSI = 15.2 + 31.1 (additional attempts) + 7.9 (increased lift force) + 26.2 (additional operators) + 4.9 (external laryngeal pressure) + 11.4 (alternative techniques) + 3.5 (Cormack grade 1) (*r* = 0.812; *r*^2^ = 0.66). When we subtracted 15 seconds from the DSI and reran the model, minimal changes were observed in the regression coefficients (Table 2).

**Table 2:** Regression coefficients of the multiple linear regression models among factors of IDS and duration of successful intubation.

	Duration of successful intubation	
	Multiple linear regression models	Mixed effects models
Factor of IDS	β ± SE	β ± SE
Constant	15.2 ± 0.6	15.7 ± 0.8
Additional attempts	31.1 ± 1.1	30.9 ± 1.1
Additional operators	26.2 ± 3.3	26.9 ± 3.3
Alternative techniques	11.4 ± 3.8	11.4 ± 3.8
Increased lift force	7.9 ± 1.2	8.1 ± 1.2
External laryngeal pressure	4.9 ± 0.9	5.1 ± 0.9
Cormack grade – 1	3.5 ± 0.7	3.2 ± 0.7
Vocal cord adduction	–	–
*R* ^2^	0.66	–
Residual variance	–	155.8

The duration of successful intubation served as the dependent variable, and common IDS factors were the independent variables. Because 12 anaesthesiologists performed a total of 1095 intubations, we reran the mixed-effects models to adjust for repeated measurements. DSI = duration of successful intubation, IDS = intubation difficulty scale, SE = standard error of the mean, SE = standard error of the mean.

The 12 anaesthesiologists performed 1095 intubations. Mixed-effects models were constructed to adjust for repeated measurements. When the intubation performer was set as the random effect, the resulting mixed-effects model was similar to the previous model, except that the extra time to intubation due to alternative techniques decreased from 11.4 to 6.9 seconds. When an additional 12 DI prediction factors were entered into the model, the results did not change (Table 2).

Perceived DI was found to be significantly correlated with IDS score and grade, with correlation coefficients of 0.75 and 0.52 (*P* < .001 in both cases), respectively. The linear regression model of the IDS factors and PDI revealed that 5 of the 7 IDS factors contributed to the model, namely increased lift force, additional attempts, additional doctors, alternative techniques, and Cormack grade ≥3 (*r*^2^ = 0.64). The need for an additional operator was the only factor that decreased perceived difficulty grades (Table 3).

**Table 3:** Regression coefficients of the multiple linear regression models among factors of IDS and perceived of intubation difficulty.

	Perceived of intubation difficulty	
	Multiple linear regression models	Mixed effects models
Operative factor	β ± SE	β ± SE
Constant	1.0 ± 0.2	0.98 ± 0.04
Additional attempts	0.3 ± 0.04	0.28 ± 0.03
Additional operators	−0.5 ± 0.1	−0.5 ± 0.1
Alternative techniques	0.4 ± 0.1	0.4 ± 0.1
Increased lift force	0.6 ± 0.04	0.5 ± 0.04
External laryngeal pressure	–	0.06 ± 0.03
Cormack grade – 1	0.42 ± 0.2	0.41 ± 0.02
Vocal cord adduction	–	–
*R* ^2^	0.64	–
Residual variance	–	0.147

Perceived intubation difficulty (grade 1–4) served as the dependent variable and common IDS factors were the independent variables. Because 12 anaesthesiologists performed a total of 1095 intubations, we reran the mixed-effects models to adjust for repeated measurements. IDS = intubation difficulty scale, PDI = perceived difficulty of intubation, SE = standard error of the mean.

## 4 Discussion

With real-world experiences of intubation in 1095 patients, we observed good correlation between DSI (*τ* = 0.584; *P* < .001) and PDI (*τ* = 0.676; *P* < .001). This finding is similar to the original IDS validation,^[10]^ which revealed that IDS was correlated with intubation time (correlation *τ* = 0.48; *P* < .0001) and VAS of difficulty (correlation *τ* = 0.63; *P* < .0001). Therefore, we revalidated the IDS as a comprehensive DI score. However, calculating the IDS score with an equal impact for every factor resulted in insufficient power to predict either DSI or PDI in clinical settings. We further quantified the average time delay or required duration for each contributing IDS factor during intubation (Tables 1 and 2) using multiple linear regression. Our analysis revealed that all IDS factors except vocal cord adduction significantly prolonged the DSI in a multiple linear regression model, with an *r* value of 0.81 and *R*^2^ of 0.66 (*P* < .001). In addition, the results were relatively consistent with the PDI of operators on 5 of the IDS contributors, with an *R*^2^ of 0.64 (Table 3). Our results support the hypothesis that IDS is a comprehensive measurement of DI. The predictive power of the regression model considering the different impacts of each contribution factor was more accurate than that of the original prediction model in which factors had equal weights. It was also better than any single predictive factor corresponding to the DI scale.

We constructed a mixed model to exclude the individual random effects from doctors and found that only the extra time to successful intubation associated with alternative techniques was different, decreasing from 11.4 to 6.9 seconds. This difference may be related to doctor preference, proficiency, and degree of familiarity with different apparatuses. The 12 anaesthesiologists achieved similar results in tracheal intubation with the classical Macintosh blade. This further demonstrates that our model is able to represent experienced anaesthesiologists performing tracheal intubation with a standard Macintosh blade in clinical practice. The robustness of the results presented in Tables 2 and 3 indicates that the prediction model is reliable and would be useful as a reference for well-trained anaesthesiologists under common tracheal intubation. This validates the IDS as an effective outcome measurement for evaluating the progress of intubation skill. The impact of each factor also helps in the formulation of crucial simulation elements during critical airway training and is useful for monitoring learning progress and providing feedback.

A substantial difference was observed between the time delays of the 6 factors affecting clinical rescue airway management. The most common manual procedures were lift force required and laryngeal pressure (40.6%) with least increased time (21.8%) of 7.9 and 4.9 seconds, respectively. Training anaesthesiologists and intubation assistants to improve laryngeal pressure and blade life force are imperative. The alternative techniques or video intubation tools required only an additional 11.4 seconds. Despite having little experience with alternative techniques, the time increase was relatively short. Additionally, rescue intubation with a video laryngoscope reportedly has a high success rate (>90%). An alternative technique may improve the quality of rescue airway management. These results indicate that training doctors in techniques other than classical Macintosh blade usage may be helpful for rescue airway management.

Cormack grade is a commonly used DI assessment. In the current study, the correlation between the Cormack grade and IDS was good (*τ* = 0.85; *P* < .001). Cormack grade is the main factor of IDS during direct laryngoscopic intubation. All patients with Cormack grades 1 to 2 were intubated within 3 attempts, with an IDS score lower than 5. The correlation between Cormack grade and DSI or PDI was moderate but significant (*τ* = 0.48 and 0.67, respectively; *P* < .001). With a Cormack grade of 3 and 4, the IDS score exceeded 5 in 9.5% and 23.5% of patients, respectively. However, 90.5% and 76.5% of patients with Cormack grades 3 and 4, respectively, were successfully intubated by experienced anesthesiologists during the first attempt. This indicates that the Cormack grade has a high sensitivity but low specificity on the DSI. Our results suggest that Cormack grade is a critical element of DI; however, the linear regression model of IDS contributing factors would be a more accurate measure than simply using the Cormack grade as an index for tracheal intubation difficulty.

The DSI had a time effect of approximately 3.5 seconds when the Cormack grade increased from grade 2 to grade 4. Patients with Cormack grades 2, 3, and 4 exhibited increased DSI durations of 3.5, 7, and 10.5 seconds, respectively. The calculated DSIs were 18.6, 22.1, and 25.6 seconds, respectively. However, the real DSI corresponding to Cormack grades 1 to 4 were 17.7 ± 13.3, 23.3 ± 11.8, 46.3 ± 38.2, and 55.1 ± 53.1 seconds, respectively. This increase may be attributed to more than 50% and more than 70% of patients with a Cormack grade greater than 3 requiring increased lift force and laryngeal pressure, respectively. In patients with a Cormack grade >2, the lift force necessary and laryngeal pressure may be combined.

Vocal cord adduction did not reach any statistically significant difference in the operating room scenario, which could be at least partially explained by the fact that almost all of the patients in the operating room were paralysed with a muscle relaxant. Only 19 of the 1051 (1.8%) patients were intubated with vocal cord adduction. Our DSI data were collected prospectively by 2 independent, nonpractitioner research assistants with stopwatches, and we applied mixed-effects modelling to control for the possible random effects of 12 anaesthesiologists and different analytic strategies. Because of the aforementioned 2 reasons and our robust results, we tentatively conclude that the relative magnitudes of time-delay effects due to each factor were quantified appropriately and would be useful for the prediction of time delay under different combinations of risk factors.

Our validation cohort had limitations. This was a single-centre observational study. Although the validation data have similar DSI (their median DSI was 22 seconds and ours was 23.9 seconds) to those used in the original study of Adnet et al, but some differences were noted. In our study, only 37% of the participants had an IDS score of 0, which was lower than that in Adnet original report (53%).^[8]^ Furthermore, the successful intubation at the first attempt was different between their study (89%) and ours (95.2%), and our cohort had more female participants. Notably, the percentage of patients with IDS scores >5 (moderate to major difficulty) was 1.8% lower in our cohort than in the original and validation reports (6.3% and 7.7%, respectively).^[8,10]^ The IDS range was much narrower in our cohort (0–10) than in the original cohort (0–20). Intubations with moderate to major difficulty also decreased dramatically between the original study and our study. Much has changed in the 20-year interval between our study and that of Adnet et al.^[8,10]^ New video intubation apparatuses were used during difficult rescue airway management.^[19,20]^ The video intubation system was used in 6.1% (3/49) of second attempts, 21.2% (4/19) of third attempts, and 58.3% (7/12) of fourth attempts or more. In our clinical practice, a video intubation system is principally used after 3 attempts. The delay in using the video intubation system until more than 3 attempts had failed in our cohort (0.8%) was similar to that reported from Duke University in the early stage (2002) but longer than that reported in a later period (2016).^[1]^ Early introduction of the video intubation system to rescue when the first attempt failed may help to improve airway management quality.^[21]^ The other possible reasons for the lower rate of “easy” intubation may be related to Taiwanese culture: Taiwanese people tend to refrain from saying something is easy.

Furthermore, anesthesiologists in Taiwan typically treat up to 1500 patients per year, with more than 80% receiving general anaesthesia, and all the anaesthesiologists in our study were highly experienced in clinical anaesthesia. Additionally, the classical Macintosh blade was almost exclusively used for the first intubation attempts. However, the original report used certified anaesthesiologists or certified nurses with more than 2 years of experience and senior (more than 3 years of full-time experience) and junior doctors.^[8]^ However, the frequency of intubations requiring more than 3 attempts was similar to the earlier stage (2002–2006) and higher than that of modern practice (after 2012),^[1]^ suggesting that simply increasing clinical practice experience may have limitations. Hospitals should be encouraged to establish a rescue strategy and consider the earlier use of the video intubation system.^[21]^ Furthermore, simulation training may improve the quality of airway management.

The current detailed analysis provides a robust guide for how each IDS contributor affects the time delay (Table 2). Six of the 7 IDS contributors were found to have independent effects on DSI. According to the mixed-effects model results, each additional intubation attempt would increase the DSI by approximately 31 seconds on average, and each additional operator would increase the DSI by 26 seconds (Table 2). The time delay predicted by each contributing factor provides a suitable reference for clinicians to establish a tailored DI strategy for each individual. For instance, 2 intubation attempts requiring a total of 46.3 seconds might be safe in an average patient breathing room air before apnea, but 3 attempts would take over 1 minute (78.1 seconds), leading to oxygen desaturation in those without preoxygenation. This is consistent with the current clinical cut-off point for DI of 60 seconds^[13]^ and 2 or 3 or more intubation attempts.^[1,6,22]^ It is also similar to the Difficult Airway Society Guidelines, which suggest that after 3 failed attempts using Plan A, clinicians should consider implementing plan B^[23]^ because the duration after the third attempt would exceed 60 seconds. Oxygen saturation in patients without preoxygenation drops to 95% within 30 seconds and to 90% within an additional 20 seconds.^[24]^ Thus, more than 50 seconds must have elapsed for oxygen saturation to drop to dangerous levels. Consideration of the effect of each contributor helps to analyse and measure the steps required for intubation in critical airway management. This prediction model may facilitate the development of strategic plans for critical airway management.

Five of the 7 IDS-contributing factors were significantly associated with the PDI (Table 3). The good correlation between the IDS contributing factors and the DSI and PDI indicates that the IDS and its factors comprehensively represent intubation difficulty. However, employing an additional operator was the only negative factor associated with the IDS. Four of the 5 independent IDS-contributing factors were identified as the elements creating difficulty in PDI, but the additional operators reduced the subjective feelings of difficulty or anxiety in the main operator. This seems to correspond with the aforementioned “calling for help” when intubation fails, which is recommended in guidelines for critical airway management.^[5,23,25]^

In conclusion, our study confirms that the IDS is a comprehensive score, and the modified IDS regression model considering the effect of each contributing factor provides a clinically useful reference for the time delay of each factor. This reference may expand the usefulness of the original IDS from simply a quantitative score to a guide to define strategies for DI in clinical and simulated settings.

## Author contributions

**Conceptualization:** Yi-Seng Tsai, Chia-Chih Alex Tseng.

**Data curation:** Ting-Wei Kang, Jung-Der Wang, Yi-Seng Tsai, Chia-Chih Alex Tseng.

**Formal analysis:** Jung-Der Wang.

**Funding acquisition:** Chia-Chih Alex Tseng.

**Investigation:** Ting-Wei Kang, Yi-Seng Tsai, Chia-Chih Alex Tseng.

**Methodology:** Jung-Der Wang, Yi-Seng Tsai.

**Supervision:** Jung-Der Wang, Chung-Ren Lin, Chia-Chih Alex Tseng.

**Validation:** Ting-Wei Kang, Chung-Ren Lin.

**Visualization:** Chung-Ren Lin.

**Writing – original draft:** Ting-Wei Kang.

**Writing – review & editing:** Ting-Wei Kang, Jung-Der Wang, Yi-Seng Tsai, Chung-Ren Lin, Chia-Chih Alex Tseng.
